# A global occurrence database of the Atlantic blue crab *Callinectes sapidus*

**DOI:** 10.1038/s41597-021-00888-w

**Published:** 2021-04-16

**Authors:** Giorgio Mancinelli, Roberta Bardelli, Argyro Zenetos

**Affiliations:** 1 Department of Biological and Environmental Sciences and Technologies (DiSTeBA), University of Salento, SP Lecce-Monteroni, 73100 Lecce, Italy; 2 National Research Council (CNR), Institute of Biological Resources and Marine Biotechnologies (IRBIM), Via Pola 4, 71010 Lesina, (FG) Italy; 3 CoNISMa, Consorzio Nazionale Interuniversitario per le Scienze del Mare, Piazzale Flaminio 9, 00196 Roma, Italy; 4 Department of Earth and Marine Sciences, University of Palermo, Via Archirafi 22, 90133 Palermo, Italy; 5 Institute of Marine Biological Resources and Inland Waters, Hellenic Centre for Marine Research, 46.7 km Athens Sounio ave., 19013 Anavyssos, Attiki Greece

**Keywords:** Biogeography, Invasive species, Macroecology

## Abstract

The Atlantic blue crab *Callinectes sapidus* is a portunid native to the western Atlantic, from New England to Uruguay. The species was introduced in Europe in 1901 where it has become invasive; additionally, a significant northward expansion has been emphasized in its native range. Here we present a harmonized global compilation of *C. sapidus* occurrences from native and non-native distribution ranges derived from online databases (GBIF, BISON, OBIS, and iNaturalist) as well as from unpublished and published sources. The dataset consists of 40,388 geo-referenced occurrences, 39,824 from native and 564 from non-native ranges, recorded in 53 countries. The implementation of quality controls imposed a severe reduction, in particular from online databases, of the records selected for inclusion in the dataset. In addition, a technical validation procedure was used to flag entries showing identical coordinates but different year of record, in-land occurrences and those located close to the coast. Similarly, a flagging system identified entries outside the known distribution of the species, or associated with unsuccessful introductions.

## Abstract (summary)

**Table:** 

Measurement(s)	geographic location
Technology Type(s)	digital curation
Factor Type(s)	location • year of record
Sample Characteristic - Organism	Callinectes sapidus
Sample Characteristic - Environment	marine biome
Sample Characteristic - Location	Globe

Machine-accessible metadata file describing the reported data: 10.6084/m9.figshare.14077112

## Background & Summary

Biological invasions are currently acknowledged as one of the main threats to the integrity of marine ecosystems^1,2^. European seas provide an impressive illustration of the extension of the phenomenon, with over 850 established non-indigenous species (NIS) since 1950^3,4^. Considerable efforts are currently made worldwide to collect data on bio-ecological traits of marine NIS to predict their invasiveness, identify introduction pathways, evaluate the risks connected with their introduction, and implement appropriate mitigation procedures^5–10^. However, the collation of information on the spatial distribution of marine NIS in invaded as well as in native ranges remains a mandatory stepping-stone in the development of effective control and management actions as well as for macroecological, evolutionary, and bioclimatic modelling studies^11–15^.

To date, a burgeoning amount of point-occurrence data have been mobilised online *via* international data-sharing networks such as the Global Biodiversity Information Facility (GBIF, www.gbif.org). They integrate information of varying quality, often compiled and verified at different times and places, while geographical and taxonomic biases may greatly challenge the robustness of the data provided^16–18^. In addition, online sources may be incomplete due to e.g., delays in updating the records, and the retrieval of additional information from “classical” sources such as published literature may result necessary to increase the completeness of the data. Thus, the limitations weakening the reliability of the three basic dimensions of species distribution data, i.e. taxonomy, space, and time, may be exacerbated by the collection of records from single sources or, when multiple sources are used, by their unsupervised collation.

In the present study, we provided a global compilation of occurrences of the Atlantic blue crab *Callinectes sapidus* Rathbun, 1896 (Brachyura: Portunidae). The species is native to the western Atlantic Ocean from Uruguay to Nova Scotia^19,20^, where it represents a commercially valuable shellfish product^21^. It is euryhaline, with a life cycle taking place in both brackish and marine habitats. In general, adult and juvenile *C. sapidus* inhabit estuaries, lagoons and other coastal environments^22^. They have omnivorous predatory habits^22–24^, and play a key functional role in regulating trophic cascades on primary producers and in controlling fluxes of energy and elements between the benthic compartment and the water column^25–27^. After mating, females migrate to near-shore marine waters to spawn; developing larval stages shift from a planktonic to a benthic life style and return to brackish habitats, where they reach maturity^22^.

*Callinectes sapidus* is native to the Western Atlantic, but it has been introduced, accidentally or intentionally, into both Asia and Europe^19^. Specifically, the species was recorded in Europe for the first time in 1901 on the Atlantic coast of France^28^, probably introduced by ballast waters^29,30^. In the Mediterranean Sea, *C. sapidus* appeared in 1947, but it may have arrived as early as the ‘30 s in the Aegean Sea^30,31^. Since then, the blue crab has greatly expanded its range in the Black and the Mediterranean Sea^32–34^ (where it is considered invasive^35,36^, thus recognized to determine adverse effects on environmental quality with negative economic and social consequences^37^), and along the European coasts of the Atlantic Ocean^38–40^. Noticeably, a significant northward expansion has been suggested also in its native range, likely triggered by sea water warming^20^.

*Callinectes sapidus* is rapidly shifting its distribution worldwide, but to date no comprehensive dataset of occurrences is available against which to verify and understand current and future variations of its distribution. Here, as a part of a risk assessment of *C. sapidus* in European waters funded by the European Commission (Project 07.0202/2019/812602/ETU/ENV.D.2), we integrated occurrence records of the species collated from online open-access biodiversity databases as well as from unpublished and published literature sources. Automatic and manual control procedures were implemented to harmonize the data and increase their quality while reducing their spatial redundancy, ultimately producing a standardized georeferenced global dataset containing 40,388 records. A validation procedure was implemented to flag occurrences showing identical coordinates but different year of record, those occurring under fully marine conditions and those located in land. Similarly, a flagging system identified entries outside the known distribution of the species, or associated with unsuccessful introductions, ultimately providing valuable selection tools for future uses of the dataset in e.g., habitat suitability investigations.

## Methods

### Open-data compilation

Occurrences of *Callinectes sapidus* in native and invaded habitats were retrieved on July 22^nd^, 2020 from the Global Biodiversity Information Facility (GBIF, www.gbif.org), Biodiversity Information Serving Our Nation (BISON, https://bison.usgs.gov), and the Ocean Biogeographic Information System (OBIS, www.obis.org). GBIF and BISON data comprised records from the citizen science initiative iNaturalist (https://www.inaturalist.org/). Citizen science, i.e. the involvement of volunteers in science, is making substantial contributions to large-scale international biodiversity monitoring^41,42^ and, as rapid flow of information on the occurrence of species is critical to implement effective monitoring actions, it also provides a precious opportunity to improve the information available on the distribution of NIS^43–45^. Acknowledging this view, we retrieved iNaturalist occurrences with no copyright for any use and without any restriction recorded between July 10^th^ and 22^nd^, 2020, as the most recent iNaturalist records included in GBIF and BISON dated July 9^th^, 2020 and July 21^st^, 2018 respectively.

**Fig. 1:**
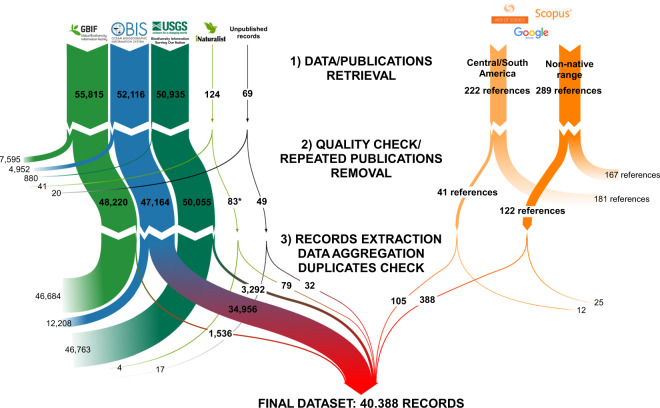
Flow chart of literature search and data extraction. If not stated otherwise, numbers in the chart generally indicate the number of records. The asterisk indicates that after quality control, in iNaturalist occurrences were included 82 “research-grade” records plus one “needs-ID” record (Source ID = 1595170075) identified as *Callinectes sapidus* by the authors after examination of the information and photographic documentation provided in iNaturalist.

The procedure adopted for the collation of records from the different online sources was implemented in the R environment^46^ and it is summarized in Fig. 1. A total of 55,815, 50,395, 52,216, and 124 records were retrieved from GBIF, BISON, OBIS, and iNaturalist respectively, using the function *occ* in the package *spocc*^47^.

A quality check was performed on the four datasets to remove entries providing no year of record. Subsequently, invalid occurrences having no, or identical, or 0,0 coordinates were excluded using the functions *cc_equ*, *cc_val*, and *cc_zero* in the package *Coordinate Cleaner*^48^. Even though records of specimens preserved in museums or in other biodiversity facilities were generally eliminated together with entries having no coordinates, the function *cc_inst* was run to identify occurrences located in a radium of 100 m around each institution. One record referring to a specimen preserved since 2015 at the Northeastern University Marine Science Center in Nahant, Massachusetts, USA, was identified in the OBIS dataset (ID# a87a5c8d-11ec-4fc4-81ee-eba9959bafc4, 42.418698°N, −70.9074°E) and excluded.

Occurrences recorded with an inadequate spatial resolution (e.g., those reported in the field “coordinatePrecision” in GBIF with an accuracy of 0.01 decimal degrees or lower) were successively eliminated by removing entries whose coordinates were reported with less than three decimals, thus with an accepted indeterminacy of approximately 100 m. In addition, iNaturalist records included in both GBIF and BISON have exclusively a “research grade” status (RG hereafter), i.e., they are subjected to a Data Quality Assessment and are georeferenced with an advanced precision. Moreover, photographs are typically included in observations in order to be validated^49^. Consensus among at least two thirds of identifiers ultimately elevates a record to “research grade”. We adopted an identical selection criterion, thus only RG iNaturalist records were selected, with one exception: the record 53602467 (dating 19/07/2020, and located 40.28892°N, 16.777°E in the Basilicata Region, Southern Italy) was classified as “needs_id”, i.e., lacking a final agreement by at least 2/3 of the members of the iNaturalist community on its identification. The record was ultimately incorporated after a thorough comparison with other records in the area and examination of associated images.

### Unpublished data collection

An effort was made to include in the dataset unpublished *C. sapidus* occurrences recorded in European waters. Specifically, 32 occurrences were directly under the authors’ possession or were obtained from authors of papers reporting on *C. sapidus* in non-native ranges by asking them for additional suggestions. 24 of them were selected, as they were complemented by photographic material allowing an unquestionable taxonomic identification and had the required level of temporal and spatial resolution. In addition, 37 occurrences were obtained from available-on-request databases either directly maintained by the Hellenic Centre for Marine Research (HCMR; https://www.hcmr.gr), or connected with the HCMR, such as the Ellenic Network on Aquatic Invasive Species^50^ (ELNAIS; https://elnais.hcmr.gr). The taxonomic reliability of these records is verified by in-house experts before inclusion in HCMR and ELNAIS databases; they were reduced to 25 after removing entries providing no or inadequate temporal and spatial information.

### Literature data extraction

A preliminary inspection of the occurrences obtained from online databases and from unpublished data indicated i) a high disproportion of records from North America as compared with Central and South America. Even though *Callinectes sapidus* reaches the highest abundances in North America from Texas to Massachusetts, the native distribution range of the species extends almost continuously from Nova Scotia and Maine to northern Argentina, including Bermuda and the Antilles^30,51^ and ii) a paucity of data from non-native ranges, as the distribution of *C. sapidus* in the last decade has remarkably expanded in the Mediterranean and Black Sea, as well as along the Atlantic coasts of Europe^32–34,40,52^. In order to fill these voids, the online databases ISI Web of Science and Scopus were searched for publications by a multiple search criterion using the term “*Callinectes sapidus*” in conjunction with the names of all Central and South American countries bordering the Atlantic Ocean, from “Mexico” to “Argentina”, in the title, abstract, and keywords. The results were supplemented with those obtained from queries on Google Scholar (https://scholar.google.com/) and saved using the freeware Harzings’s Publish or Perish ver. 7.27.2849^53^, using identical keywords together with the corresponding terms in Spanish or Portuguese (e.g., “jaiba azul”, cangrejo azul”, “siri azul”) to have access to additional documents written in other than the English language.

For European non-native areas, the information presented in the reviews by Nehring^30^ and Mancinelli *et al*.^32^ were supplemented with data obtained by a search on ISI Web of Science and Scopus using the term “*Callinectes sapidus*” in conjunction with “invasive”, “non indigenous”, and “alien” in the title, abstract, and keywords. They were further complemented with information obtained from Google Scholar as already described. Literature data searches were completed by August 13^th^, 2020, to meet the time deadlines of the project motivating the study while allowing for an in-depth examination of the retrieved information and their integration with those obtained from other sources.

The literature search for *C. sapidus* occurrences in Central and South America resulted in a total of 181 articles, 84 obtained from Scopus published between 1975 and 2020 and 97 from Web of Science published from 1990 to 2020. They were reduced to 140 after duplicates removal (Fig. 1). Subsequently, the titles and abstracts were screened and those performed in the United States and laboratory investigations were excluded. The remaining 72 eligible full-text articles were examined in detail to filter those where *C. sapidus* occurrence was reported explicitly, together with information on the country, latitude and longitude, and at least the year of the record. Publications where the record had no coordinates but was reported in maps were also included in the selection. An identical procedure was adopted for the 182 documents identified in Google Scholar. 32 articles were eventually selected, together with nine sources identified on Google Scholar. For publications were the records had no coordinates but were reported in maps, all contextual information was used to locate the geographic area of the study in Google Earth. Maps were extracted using the freeware GIMP (ver. 2.10.20, https://www.gimp.org/), overlaid to the study area in Google Earth, and adjusted to match the background. Subsequently, occurrences were georeferenced using placemarks and recorded. A total of 105 individual records were extracted (Fig. 1).

The literature search for occurrences in non-native areas resulted in 75 articles from Scopus published between 2002 and 2020, and in 122 articles from Web of Science published between 2001 and 2020. 92 more references published between 1901 and 2020 were identified in Google Scholar or checking the literature sources cited in the reviews used as references. After removing duplicates, articles were examined in detail to select those reporting adequate spatial and temporal information on the records and reduced to 122. Subsequently, a total of 395 occurrences were extracted (Fig. 1).

### Dataset final collation

All the records obtained from the different sources were eventually collated and checked for duplicates (Fig. 1). Specifically, to exclude identical data points that may have been geo-referenced slightly differently, the function *cc_dupl* was used to eliminate entries showing identical coordinates to the third decimal degree (accepted indeterminacy approx. 100 m) and the same year of record.

## Data Records

### General considerations

Once subjected to the quality control procedures, the final dataset consisted of 40,388 records of *Callinectes sapidus*, 39,824 from native and 564 from invaded areas (Fig. 1, Table 1), and it is publicly accessible for download from a permanent repository (10.6084/m9.figshare.12896309^54^). The dataset includes fields reporting taxonomic information, the World Register of Marine Species (WoRMS; www.marinespecies.org) unique identifier (aphiaID), and provides information on geographical location (e.g., coordinates in decimal degrees), reference to original sources, as well as the flagging system implemented (Table 2). Specifically, the set of included flagging fields allows users to subset the dataset considering i) the native or non-native status of the species, as well as ambiguous identifications and unsuccessful intentional introductions outside the native range; ii) the country where the records are located, and whether they occur in marine, coastal, or in-land areas, and iii) only records characterized by different coordinates, or alternatively, only a single location for which e.g., the temporal variation in the occurrence of *C. sapidus* can be examined. In addition, the dataset is complemented by a complete list of the cited literature sources, including permanent identifiers (bibliographic Citation DOI) if available, and a list of notes numbered according to the flags in the field “noteID”.

**Table 1:** Summary of the 43,388 records included in the dataset, considering the status (native vs. non-native) and location (marine vs. coastal vs. in-land; see text for further details).

Status		BISON	OBIS	GBIF	iNaturalist	Other sources	Total
Native	**Overall**	**3,291**	**34,948**	**1,407**	**73**	**105**	**39,824**
	Marine (Flag “TRUE”)	1,929	24,218	544	19	68	26778
	coastal (Flag “FALSEcoast”)	1,088	8,241	723	40	33	10125
	in-land (Flag “FALSE”)	274	2,489	140	14	4	2921
Non-native	**Overall**	**1**	**7**	**130**	**6**	**420**	**564**
	Marine (Flag “TRUE”)	1	5	51	2	197	256
	coastal (Flag “FALSEcoast”)	—	1	69	2	174	246
	in-land (Flag “FALSE”)	—	1	10	2	49	62
**Total**		**3,292**	**34,955**	**1,537**	**79**	**525**	**40,388**

The category “Other sources” includes records collated from personal communications and literature sources identified using Scopus, Web of Science, and Google Scholar.

**Table 2:** Description of the fields used in the dataset.

Field	Description
recordID	A progressive number univocally identifying each record.
scientificName	Scientific name of the species, including the name of who described the taxon originally.
aphiaID	Unique identifier of the species provided by the World Register of Marine Species (WoRMS; www.marinespecies.org).
source	The source of the record. For literature sources the name of the author and the publication date is provided; for sources with more than two authors the abbreviation “*et al*.” is used. Personal communications are reported citing the name of the provider followed by “pers. comm.”.
sourceID	The identification code originally provided for the record by online databases; “NA” if not available.
day	The two-digit day in which the record occurred; “NA” if not available.
month	The two-digit month in which the record occurred; “NA” if not available.
year	The four-digit year in which the record occurred; “NA” if not available.
decimalLatitude	Geographical latitude in decimal degrees of the record location.
decimalLongitude	Geographical longitude in decimal degrees of the record location.
country	Country in which the record occurred.
status	Status of the species in the country where it was recorded; “NAT” if native, “NIS” if non-native.
noteID	Additional comments on non-native records related with unsuccessful human introductions or to dubious identifications. Numbered by integers, they refer to notes deposited as a separate text file together with the database and the list of reference publications in the data repository (10.6084/m9.figshare.12896309^54^). “NA” if not available.
uniqueness	Flag = “TRUE”: the most recent records with different coordinates to the third decimal; = “FALSE”: the remaining records.
uniquenessID	Additional variable flagging each entry with unique coordinates (flag = “TRUE” in variable “uniqueness”) with a progressively increasing integer, while less recent records in the same location are flagged by the same integer;
sea	Flag = “TRUE”: records occurring under fully marine conditions; flag = “FALSE” for in-land records; flag = “FALSEcoast” for in-land records occurring in coastal areas covered by Bio-ORACLE raster layers.

An overall examination of the data summarized in Table 1 indicates that the procedure of collation of information from multiple online databases as well as unpublished and literature sources implemented in the present study was effective in building a comprehensive global dataset of occurrences of *C. sapidus*. Indeed, if used alone the four databases were inadequate in providing the entries that were eventually included, in particular for non-native areas, where unpublished data and the published literature made a remarkable contribution of records. In addition, it is apparent that some databases contributed a substantial number of entries only for native areas, while others prevailed for non-native ranges (i.e., OBIS vs. GBIF). This indicates that, at least in the case of *C. sapidus*, depending on the ultimate scope motivating the collation of the data some online databases may result more comprehensive than others. In contrast, the quality of the included data, in terms of both spatial and temporal standardization, together with the highly functional selection tools provided by the flagging fields, make the present dataset a fine-tuned, comprehensive baseline for e.g., future habitat suitability investigations in both native and non-native ranges of the species.

### Data sources

The dataset collated information from four distinct online repositories, 160 literature sources, and 12 unpublished databases and personal communications. In general, the majority of records resulted from online repositories (Table 1; 98.7% of the records) in particular from OBIS (86.5%) followed by BISON, GBIF, and iNaturalist (8.1, 3.8, and 0.2%, respectively).

For native ranges, data obtained from on-line repositories outnumbered other sources (99.7%); of these, 7.3% were in-land data points, while a considerable contribution was provided by coastal records (25.4%). In-land occurrence points were 3.8% of the total obtained from literature/personal communication sources, and coastal records contributing for 31.4%. In contrast, literature sources provided a major contribution of records in non-native areas (74.5%). Only 11.7% of these data points were located on land, while 41.4% of them occurred in coastal areas.

### Spatial and temporal coverage

**Fig. 2:**
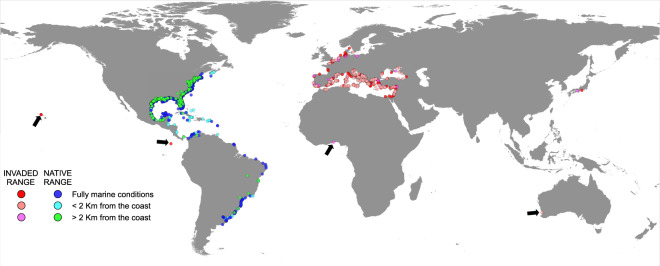
Global dataset of occurrences of the Atlantic blue crab *Callinectes sapidus* in native (in blue) and non-native distribution ranges (in red). For the sake of clarity, Antarctic areas are omitted. Records located under fully marine conditions are indicated, while those located on land are reported differentiating those occurring in coastal areas (see text for details). Arrows indicate non-native records testifying unsuccessful human introductions or doubtful identifications.

Occurrences in native habitats (Fig. 2) were located between a latitude of 38.59159°S, recorded in Argentina in 1963 (recordID# 40206) and 44.647793°N, recorded in Canada in 2019 in Nova Scotia (recordID# 862). The recent Nova Scotia record is located at a higher latitude than that verified by Johnson^20^ in 2014, who suggested a northward expansion of the historic distribution range of *Callinectes sapidus* along the eastern coast of the United States towards the Gulf of Maine. Indeed, three more occurrences in Nova Scotia recorded between 2019 and 2020 (recordID# 707, 347, and 281) support the hypothesis. The record in Argentina suggests in first instance that the expansion of the blue crab distribution range may be limited to the northern hemisphere. it is worth noting, however, that a number of occurrences in Argentina between 36.292818°S and 36.786104°S have been recorded from 2018 to 2020 (e.g., recordID# 1083 and 90). Thus, the data herein presented may suggest that a range shift in the distribution of the species along the western Atlantic coasts is occurring in both hemispheres. However, more detailed macroecological investigations are needed to corroborate the hypothesis, which may benefit from the present database for implementing e.g., environmental niche modelling approaches.

**Fig. 3:**
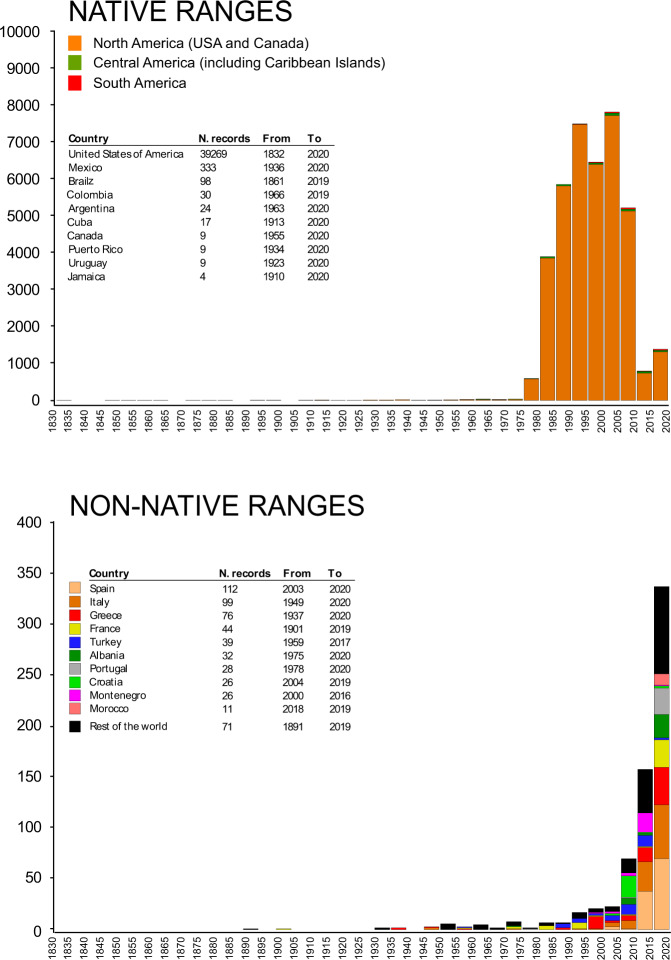
Records of the Atlantic blue crab *Callinectes sapidus* per year in native (top) and invaded ranges (bottom). In the inserts, the first ten countries per number of occurrences are reported, together with the first and last year of record of the species.

The temporal distribution of native records covered 188 years, from 1832 to 2020 (Fig. 3). The frequency distribution showed a bell-shaped pattern, with most of the occurrences recorded in the period comprised between 1990 and 2010. The majority of the data points were from the United States of America, which showed a number of records two orders of magnitude higher than Mexico and Brazil (Fig. 2). Noticeably, a significant aliquot of Brazilian records and, to a minor extent, of those from Colombia and Argentina were from literature sources (Brazil: 67.3%; Colombia: 40%; Argentina: 30%). On the other hand, online repositories provided most of the data points for Mexico (96.4%), further confirming that the efforts made in the present study to integrate data from multiple sources allowed the collation of a dataset with advanced characteristics of geographic coverage of the global distribution of *Callinectes sapidus*. Conversely, it is apparent that each source, taken alone, is ineffective in providing accurate information on the actual occurrence of the species in Central and South America.

The distribution of the 564 data points in non-native ranges spanned from 1891 to 2020, with a nearly exponential pattern after 2000–2004 (Fig. 3). Intriguingly, the pattern may be a result of both an effective increase in the number and spatial distribution of established populations in non-native areas, as well as an increase of the awareness of the species in the scientific and non-scientific community. To provide an ultimate explanation is beyond the scope of the present study; however, it is likely that the increase may actually reflect an expansion of the species, given the morphological characteristics of *C. sapidus* in terms of size and coloration and the relative ease with which it can be identified (but see the notes in the dataset for some counterexamples). Two were confirmed occurrences in Japan, while the bulk of the records (557) were from European Atlantic waters, the Mediterranean Sea, and the Black Sea. In the Atlantic (72 records), the most northward occurrences were located in Denmark on the Skagerrak at latitudes higher than those observed in native habitats (e.g., 57.71823°N, recordID# 4876), indicating that after its first record in Europe in the Gulf of Biscay^28^, *C. sapidus* has extended its range northward establishing at latitudes not reached in native habitats. Sea surface temperatures are likely to have determined such a differentiation, since at middle and high latitudes the eastern Atlantic Ocean is warmer than the western Atlantic due the effects of the Gulf Stream^55^. The expansion in European Atlantic waters is still ongoing, as testified by a number of observations from Portugal, Spain, and France made after 2017 (25, 2, and 3 records, respectively). In the Mediterranean Sea (458 records) the species has reached a condition of virtual ubiquity (Fig. 2). Spain, Italy, and Greece show the highest number of records, and most recent (Fig. 3). Spain, in particular, is characterized by an anomalous accumulation of records within a relatively limited time range (from 2003), as compared with Greece and Italy where the blue crab appeared between 1947 and 1949 (Fig. 3). Noticeably, the recent entries from Morocco, Algeria, and Tunisia (20 occurrences, all recorded after 2017) indicate that the distribution of the blue crab, mainly concentrated in northern and eastern Mediterranean sectors, is extending southward along the African coasts. The relatively low number of records from the Black Sea (15 occurrences) indicate that the general oceanographic conditions of the basin may be relatively unsuitable for the species^30^. However, some recent records in Turkey (recordID# 1289 and 1679) testify a range expansion also in the Black Sea, even though not at rates comparable with those observed in e.g., the Mediterranean.

The remaining 4 records in non-native areas were all from GBIF; they referred to unsuccessful introductions and to doubtful, not confirmable identifications. They are flagged in the dataset (field “noteID”) and are described in detail in the notes complementing the dataset itself (10.6084/m9.figshare.12896309^54^).

## Technical Validation

The quality control procedures adopted to exclude duplicate or low-quality records imposed a severe selection of the records, with the exclusion of a minimum of 33% (OBIS) to a maximum of 97% (GBIF) of the data originally downloaded from online databases (Fig. 1). Similarly, a considerable number of literature sources, including publications of potential interest given the number of records reported or the location (for example^34,56^), were necessarily excluded. In addition, four validation measures were implemented using a flagging procedure, namely:

1) identification of records in native and invaded ranges. The shapefile layer of the global distribution of the species provided by the Food and Agriculture Organization of the United Nations (http://www.fao.org/geonetwork/srv/en/metadata.show?currTab=simple&id=55166, accessed August 20^th^, 2020) was modified by adding a dichotomous field containing the attribute variables “NAT” and “NIS” identifying the native and non-native ranges of the species in the Western Atlantic and in the rest of the world, respectively. Subsequently, the layer was overlaid to the dataset once the latter was converted into a shapefile using the *st_as_sf* function in the R package *sf*^57^. The procedure allowed an automatic flagging of the entries; a manual check was further performed to verify the consistency of the location of the records with the assigned flags. The information were ultimately included in the dataset (field “status”, Table 2). Additionally, after checking the available literature material, we added a second field (“noteID”; Table 2) containing integers progressively numbering records of dubious identifications and unsuccessful intentional introductions outside the native range of the species;

2) identification of records characterized by unique coordinates. The *Coordinate Cleaner* function *cc_dupl* was used without any additional condition to automatically identify with the flag “FALSE” the records showing identical coordinates to the third decimal degree; when two (or more) occurrences had identical coordinates but different years of record the flag “TRUE” was used to identify the most recent. The information was included in the dataset in the field “uniqueness”; we added a second field (“uniquenessID”; Table 2) where each entry with unique coordinates was identified by a progressively increasing integer, while less recent records in the same location were flagged by the same integer;

3) identification of in-land records. Even though *C. sapidus* is a fully aquatic brachyuran, it is extremely euryhaline: in both native and non-native habitats the species is found under fully marine conditions at salinities up to 34‰ as well as in freshwaters as far as 195 km upstream from the coast^19,30^. The *Coordinate Cleaner* function *cc_sea* was used to identify marine and in-land records (flagged in the dataset as “TRUE” and “FALSE” in the field “sea”, respectively; Table 2). An operative corroboration of the accuracy of the classification was performed by overlying the georeferenced dataset to a stack of Bio-ORACLE^58^ raster layers, verifying that 100% of the entries classified as “true” marine records corresponded with pixels of the layers’ grid containing information. Noticeably, the procedure identified also 10,371 entries originally classified as in-land records for which parameters could be extracted from the oceanographic layers. A check performed by importing these records in Google Earth and visually verifying their actual location indicated that they generally occurred in coastal habitats such as lagoons, estuaries, and other transitional systems (*sensu*^59^), and that they were covered by Bio-ORACLE layers’ grid because of their vicinity to the coast and the resolution of the layers themselves (5 arc-minutes, corresponding to approximately 9.4 Km at the equator). Accordingly, the flag “FALSEcoast” and the term “coastal” were used to identify them in the field “sea” of the database (Table 2) and in in the remainder of the text, respectively.

## Usage Notes

Some aspects related with the use of the dataset are worth being emphasized:

1) The procedure allowed to flag fully marine records that may be particularly suitable for ecological modelling using oceanographic GIS layers included in e.g., Marspec or BIO-ORACLE^58,60^. Coastal occurrences (i.e., those flagged “FALSEcoast” in the “sea” field) may be also used with Bio-ORACLE layers but with caution, as they can be included in the analysis only as a consequence of the resolution of the layers’ grid (see above in the Technical Validation section). However, the coastal and in-land entries may also be used with climatic layers such as WorldClim^61^ or ENVIREM (https://envirem.github.io/) or freshwater-specific layers^62,63^ for modelling e.g., the environmental drivers regulating the in-land dispersion capability of *Callinectes sapidus*;

2) notwithstanding the efforts made to increase the entries from locations other than North America, the records included in the present dataset appear to be still heavily clustered (Fig. 2). Here we opted to include occurrences showing identical coordinates but different years of record, providing flagging variables (i.e., in the fields “uniqueness” and “uniquenessID”) that can be used to subset the data in order to include only records characterized by unique coordinates, or alternatively, to select a single location and to verify temporal variations in the occurrence of the species. Beside this, we did not implement a spatial thinning procedure, leaving to the users the option to perform it according to their necessities. Nonetheless, for the sake of example we used the R package spThin^64,65^ to perform a thinning process to assess the aggregation of the 23,585 unique records in native and non-native ranges. The distance between data points was set at 1 Km taking as a reference the highest spatial resolution of oceanographic layers available from Marspec^60^ (i.e., 30 arc-seconds, corresponding to an approximate distance of 1 Km) with 100 repetitions of the thinning process.

As a result, the 23,031 occurrence points in native ranges were reduced to 12,117, showing a remarkable aggregation of the records at a scale < 1 Km and indicating that nearly 47% of the entries could be considered redundant in future environmental suitability modelling studies carried out at a multi-continental scale. In contrast, the thinning procedure performed in non-native ranges produced 478 records; this corresponds to 86% of the occurrences included in the dataset (554), and indicate a relatively low degree of aggregation and potential sampling bias.

## Data Availability

There is no custom R code produced during the collation and validation of this dataset.
